# Eight years of sales surveillance of antimicrobials for veterinary use in Germany—What are the perceptions?

**DOI:** 10.1371/journal.pone.0237459

**Published:** 2020-08-10

**Authors:** Lydia M. Köper, Christoph Bode, Alice Bender, Inke Reimer, Thomas Heberer, Jürgen Wallmann

**Affiliations:** Department Veterinary Drugs, Federal Office of Consumer Protection and Food Safety (BVL), Berlin, Germany; University of Lincoln, UNITED KINGDOM

## Abstract

A surveillance system for sales volumes of antimicrobial agents for veterinary use was established in Germany in 2011. Since then, pharmaceutical companies and wholesalers have been legally obliged to report annual volumes of veterinary antimicrobial products sold to veterinary practices or clinics located in Germany. The evaluation of sales volumes for eight consecutive years resulted in a considerable total decrease by 58% from 1706 tons to 722 tons. During the investigation period, two legally binding measures to control the risk of antimicrobial resistance resulting from the veterinary use of antimicrobials were introduced, a) the German treatment frequencies benchmarking in 2014 and b) the obligation to conduct susceptibility testing for the use of cephalosporins of the 3^rd^ and 4^th^ generation and of fluoroquinolones in 2018. Both had a marked impact on sales volumes. Nonetheless, the category of Critically Important Antimicrobials as defined by the World Health Organization kept accounting for the highest share on sales volumes in Germany in 2018 with 403 tons, despite an overall reduction by 53%. Sales surveillance is considered essential for data retrieval on a global scale and inter-country comparison. However, the usability of a surveillance system based on sales data for risk management of antimicrobial resistance has limitations. The German system does not include off-label use of antimicrobial products authorized for human medicine and does not allow for identification of areas of high risk according to animal species, farm and production types and indications for treatment. For further reduction and enhanced promotion of a prudent use of antimicrobials, targeted measures would be required that could only be deducted from use data collected at farm or veterinary practice level. A surveillance system based on use data is currently lacking in Germany but will be established according to Regulation (EU) 2019/6 on veterinary medicinal products.

## Introduction

Antimicrobials are invaluable agents in combating bacterial infectious diseases in humans and animals. However, therapeutic success can be compromised due to the emergence and spread of antimicrobial resistance. Pathogenic bacterial species that acquired multidrug resistance (defined as acquired non-susceptiblity to at least one agent in three or more antimicrobial classes [1]), pose a health risk in particular. As a consequence of multidrug resistance, certain antimicrobials can become one of a few treatment alternatives or even the sole remaining therapeutic option for severe bacterial infections [2–8]. At worst, no antimicrobial treatment option is left.

The World Health Organization (WHO) classifies and prioritizes antimicrobials according to their medical importance for humans [8]. Most of the antimicrobial classes currently assigned to the category of outmost importance, the Critically Important Antimicrobials (CIA) of Highest Priority, are also authorized as veterinary medicinal products. In Germany, this applies to cephalosporins of the 3^rd^ and 4^th^ generation, macrolides, polymyxins and quinolones whereas cephalosporins of the 5^th^ generation and glycopeptides are not authorized for veterinary use. The majority of antimicrobial classes additionally ranked as CIAs but of High Priority, are solely authorized as human medicinal products in Germany. Exceptions are two antimicrobial classes that are widely used in animals, aminoglycosides and aminopenicillins. To date, only one antimicrobial class is exclusively authorized as veterinary medicinal product in Germany, the ionophores. Monensin is indicated for ketosis prevention in dairy cows. It is administered prepartum to individual animals that are at high risk for ketosis and thus considered to comprise a minor proportion of antimicrobial consumption in dairy cows. Ionophores can also be used as coccidiostats in poultry in Germany, but as such are authorized as feed additives. The use in poultry does therefore not fall under the regulatory framework of veterinary medicinal products. Antimicrobial prophylaxis may only be conducted under certain circumstances in Germany (for instance as infection prevention in surgical interventions) and is usually limited to individual treatments. The use of antimicrobials for growth promotion is prohibited in all Member States of the European Union (EU) since 2006 [9].

The One Health approach, as pursued by the German Antibiotic Resistance Strategy (DART 2020) [10], considers human, animal and environmental health as inevitably linked [11]. Antimicrobial resistance is driven by any use of antimicrobials [12–15] and is not restricted to the sector of its emergence [16–19]. Both resistant bacteria and resistance genes can be mutually transferred between humans, animals and the environment. A direct transmission from animals to humans, as described for methicillin–resistant *Staphylococcus aureus* (MRSA) and extended-spectrum ß-lactamase (ESBL)-producing *Escherichia coli* [20–22], can occur via two pathways, direct contact or via the food chain. The environment provides a potential link for indirect transmission. Hence, it is possible that resistance emerging from antimicrobial use in animals spreads to humans and affects their therapeutic success. Phenomena like co- and cross-resistance can further contribute by providing or preserving mechanisms to inactivate or eliminate additional antimicrobial agents or classes. Although the specific impact is difficult to quantify [23, 24], it is important to gain knowledge on the amounts of antimicrobials used in animals. It is estimated that 73% of all antimicrobials used globally are administered to food producing animals [25] while antimicrobial consumption in animal production is expected to increase by 67% until 2030 [26]. Such estimates are partly based on the derivation of data from little available data sets due to the absence of comprehensive surveillance programmes in a large number of countries. Relevant data would be required to counteract the predicted increase by regulatory interventions [25]. Besides targets for a reduction of antimicrobial consumption in animals, enhanced efforts in antimicrobial stewardship in the veterinary sector are required to not further drive antimicrobial resistance by an imprudent use of antimicrobials [27–31].

Until 2011, sales volumes of veterinary antimicrobials in Germany could only roughly be estimated. The latest available estimate of a total sales volume of 784 tons by the Federation for Animal Health (BfT) referred to the year 2005 [32]. It was based on extrapolated data of selected German regions that had originally been collected for market characterization. To obtain reliable and standardized data, the implementation of a surveillance system on sales volumes of antimicrobials for veterinary use was an objective of the first German Antibiotic Resistance Strategy (DART) [32]. Pharmaceutical companies and wholesalers were legally obliged to report sales volumes of veterinary antimicrobial products for the first time for the year 2011. The aim of this article is to present the development of sales volumes of antimicrobials for veterinary use in Germany according to the WHO prioritization for eight consecutive years, to examine the impact of regulatory interventions and to discuss assets and drawbacks of a surveillance system based on sales data.

## Methods

### Regulatory framework

Our study was based on the regulatory framework for the reporting of sales volumes of antimicrobial agents authorized as veterinary medicinal products in Germany which was formed by amendments of the German Medicinal Products Act [33] in 2009 and 2011, respectively, as well as by the adoption of the German ‘Regulation on the Database-supported Information System on Medicinal Products of the German Institute for Medical Documentation and Information (DIMDI-AMV)’ [34]. Pharmaceutical companies and wholesalers are obliged to annually report the volumes of veterinary antimicrobial products sold to practices and clinics in Germany that operate a veterinary dispensary to the German Institute for Medical Documentation and Information (DIMDI). Medicated premixes and human antimicrobial products (off-label use) are excluded.

### Processing of data

Processing of data was conducted as previously described by Hauck *et al*. [35]. In brief, reported data was verified by formal and content-related aspects, e.g. accordance with sales data from periodic safety update reports (PSUR). Based on Marketing Authorization Numbers (MAN) and Substance Identification Numbers (SIN), additional information on products was retrieved from the Drug Information System of the German Higher Federal Authorities (AMIS), for instance administration routes, target species and amounts of active substance.

The amount of active substance for each product was calculated as follows:

$$A_{AI}=PS\times S_{\mathrm{AI}/R}\times \Sigma \mathrm{delivered}\;\mathrm{packages}$$ (1)

A_AI_ = amount of active ingredient, PS = package size, S_AI/R_ = strength of active ingredient per reference quantity.

If active ingredients were contained in form of chemical derivatives (e.g. salts or esters), the quantity of the active moiety was calculated based on the molecular weight according to the European Pharmacopoeia (Ph. Eur.) [36]:

$$A_{\mathrm{AM}}=A_{AI}\;x\;M_{AM}/M_{\mathrm{AI}}$$ (2)

A_AM_ = amount of active moiety, A_AI_ = chemically modified active ingredient, M_AM_ = molar mass of active moiety, M_AI_ = molar mass of chemically modified active ingredient.

Sales volumes of active ingredients originating from different products were summed up and assigned to the corresponding antimicrobial classes in tons per active ingredient. Classification and prioritization was conducted in accordance with the 6^th^ revision of the WHO CIA list [8] as shown in Table 1. Three major categories were defined by WHO, Important Antimicrobials (IA), Highly Important Antimicrobials (HIA) and Critically Important Antimicrobials (CIA), corresponding to their medical relevance for humans. The category of CIAs was further divided into two subcategories, the CIAs of High Priority and the CIAs of Highest Priority.

**Table 1:** Prioritization of antimicrobial classes according to the WHO list of Critically Important Antimicrobials for human medicine.

Critically Important Antimicrobials		Highly Important Antimicrobials	Important Antimicrobials
of Highest Priority	of High Priority		
cephalosporins of the 3^rd^ generation	aminoglycosides	amphenicols	aminocyclitols
cephalosporins of the 4^th^ generation	aminopenicillins	cephalosporins of 1^st^ gen.	cyclic polypeptides
macrolides		lincosamides	ionophores*^#^
polymyxins		anti-staphylococcal penicillins	nitrofuran derivatives*
quinolones		narrow spectrum penicillins	nitroimidazoles*
		steroid antibacterials*	pleuromutilins
		sulfonamides and dihydrofolate reductase inhibitors	
		tetracyclines	

Only antimicrobial classes that are authorized as veterinary medicinal products in Germany are listed.

*: Detailed data are not presented as it would affect business and trade secrets of marketing authorization holders in Germany.

^#^: The antimicrobial class is not authorized for use in human medicine.

## Results

### Total sales volumes

Total sales volumes of veterinary antimicrobials, referring to the total amounts of active ingredient, are presented in Table 2. Initial sales volumes were determined for the year 2011 and amounted to 1,706 tons. From 2011 to 2012, sales volumes decreased by 5.08%. They descended by another 10.34% from 2012 to 2013 and 14.69% from 2013 to 2014, respectively. The consecutive year, a large reduction in sales volumes was determined, accounting for 34.97%. Since then, this development decelerated. Sales volumes declined by another 7.83% from 2015 to 2016 and by 1.23% and 1.46% from 2016 to 2017 and 2017 to 2018, respectively. In total, a decrease of sales volumes by 57.65% (983 tons) was determined for antimicrobials authorized for veterinary use in Germany from 2011 to 2018.

**Table 2:** Total sales volumes of veterinary antimicrobials in Germany.

year	volume [t]	change [%]
2011	1705.659	
2012	1619.039	-5.08
2013	1451.619	-10.34
2014	1238.340	-14.69
2015	805.281	-34.97
2016	742.258	-7.83
2017	733.108	-1.23
2018	722.430	-1.46
**total**	**-983.229**	**-57.65**

Volumes represent the total quantities of active ingredient sold in tons [t] within the given year. Changes expressed in percent relate to the sales volumes of the previous year.

### Sales volumes according to the WHO prioritization categories of antimicrobial classes

**Fig 1:**
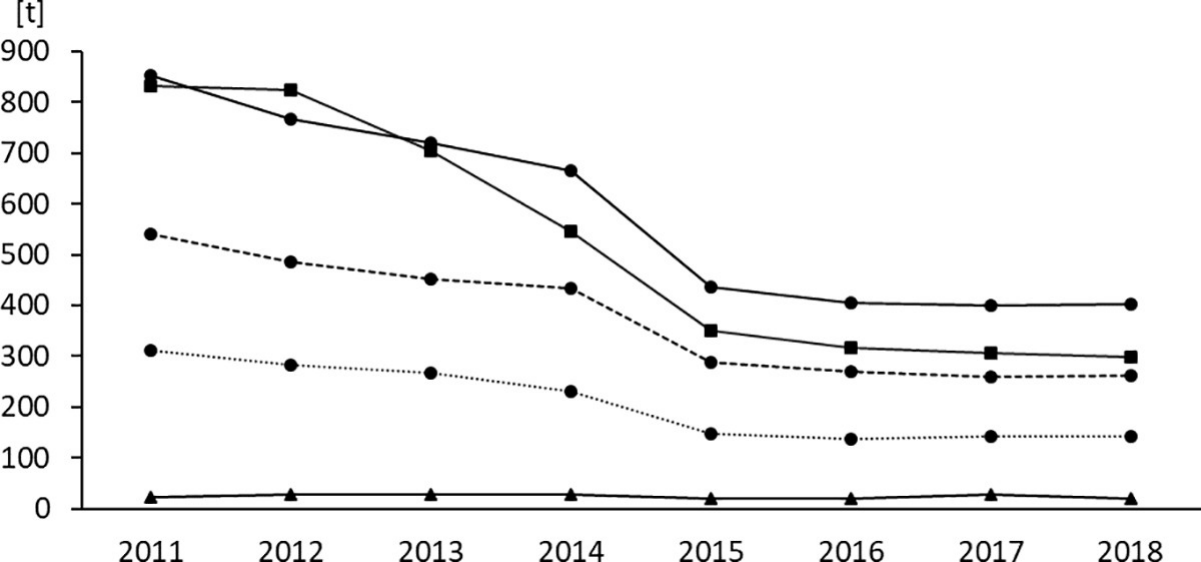
Sales volumes of antimicrobial classes prioritized according to their medical importance for humans (WHO CIA list). Sales volumes are expressed in tons [t] of active ingredient. Triangles: Important Antimicrobials (IA), squares: Highly Important Antimicrobials (HIA), circles: Critically Important Antimicrobials (CIA). Dashed line: CIA of High Priority (CIA subcategory), dotted line: CIA of Highest Priority (CIA subcategory).

Fig 1 shows sales volumes of antimicrobial classes prioritized according to the 6^th^ revision of the WHO CIA list [8]. Absolute sales volumes and percentages of change referring to the previous year are additionally presented in Table 3.

**Table 3:** Changes in sales volumes of antimicrobial classes prioritized according to their medical importance for humans (WHO CIA list).

Year	volume of IAs [t]	change [%]	volume of HIAs [t]	change [%]	volume of CIAs [t]	change [%]
2011	23.500		830.674		851.485	
2012	27.861	18.56	823.886	-0.82	767.292	-9.89
2013	28.668	2.90	703.794	-14.58	719.157	-6.27
2014	26.875	-6.26	545.143	-22.54	666.322	-7.35
2015	20.130	-25.09	349.329	-35.92	435.822	-34.59
2016	20.826	3.46	316.029	-9.53	405.403	-6.98
2017	27.831	33.62	305.607	-3.30	399.670	-1.41
2018	20.277	-27.14	298.860	-2.21	403.293	0.91
**total**	**-3.223**	**-13.71**	**-531.814**	**-64.02**	**-448.192**	**-52.64**

Volumes represent the quantities of active ingredient sold in the respective antimicrobial category in tons [t] within the given year. Changes expressed in percent relate to the sales volumes of the previous year.

**Fig 2:**
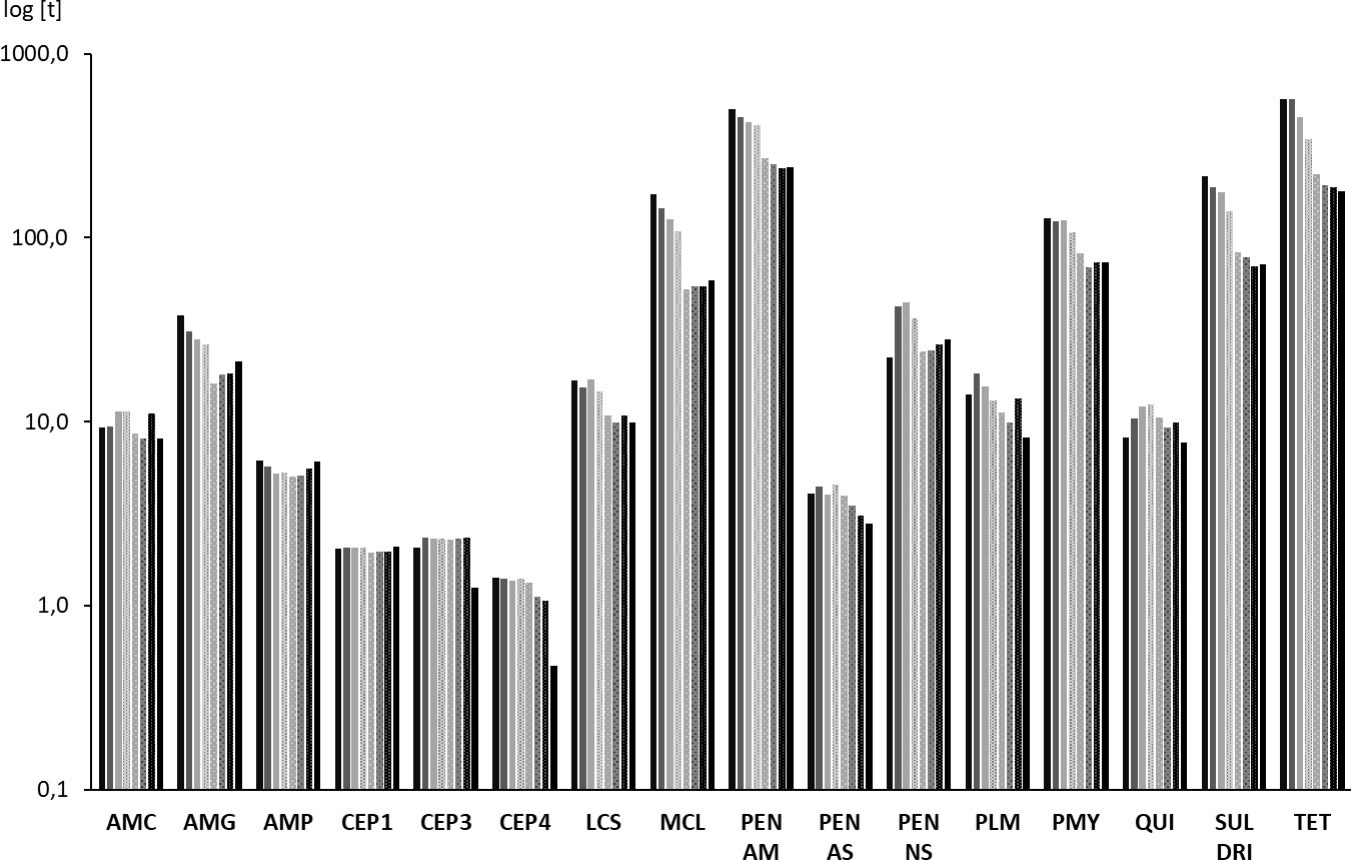
Sales volumes of 16 antimicrobial classes according to the WHO classification scheme. Semilogarithmic graph on sales volumes expressed in tons [t] of active ingredient. Bars from left to right indicate the years from 2011 to 2018. AMC: aminocyclitols, AMG: aminoglycosides, AMP: amphenicols, CEP1: cephalosporins of the 1^st^ generation, CEP3: cephalosporins of the 3^rd^ generation, CEP4: cephalosporins of the 4^th^ generation, LCS: lincosamides, MCL: macrolides, PEN AM: aminopenicillins, PEN AS: antistaphylococcal penicillins, PEN NS: narrow spectrum penicillins, PLM: pleuromutilins, PMY: polymyxins, QUI: quinolones, SUL DRI: sulfonamides and dihydrofolate reductase inhibitors, TET: tetracyclines.

Since the sales surveillance was launched, sales volumes of IAs have always been comparably low, accounting for less than 30 t. The total reduction obtained from 2011 to 2018 accounted for 13,71%. Sales volumes of HIAs initially amounted to 831 tons. Starting in 2013, they substantially reduced with the largest reduction of 196 tons (35.92%) occurring from 2014 to 2015. From 2015 to 2018, sales volumes of HIAs decreased by 14.45% in total. CIAs have been sold at highest volumes throughout the observation period with the year 2012 being the only exception thus far. Following a consistent decrease from 851 tons in 2011 to 666 tons in 2014 (21.75% reduction), sales volumes of CIAs considerably decreased by 231 tons (34.59%) from 2014 to 2015. Until 2017, they decreased by another 36 tons (8.29%). From 2017 to 2018, a slight increase by 4 tons (0.91%) was determined. If CIAs were disaggregated into the subcategories of CIAs of High Priority and CIAs of Highest Priority, both displayed a similar course (Fig 2). The main reduction in sales volumes occurred from 2014 to 2015, accounting for 148 tons (33.93%) and 83 tons (35.84%), respectively. It is noteworthy that sales volumes of CIAs of Highest Priority were slightly rising since 2016, accounting for an increase of 5 tons (3.95%) until 2018. Regarding CIAs of High Priority, a slight increase by 3 tons (1.35%) was determined from 2017 to 2018. The overall decrease in sales volumes of CIAs and in particular of the subcategory of CIAs of Highest Priority was mainly attributable to a pronounced reduction of macrolides. In general, the development of sales volumes reached a stage of saturation at a significantly lower level compared to the initial situation recorded in 2011.

### Sales volumes according to antimicrobial classes

Antimicrobial agents with similar modes of action were grouped into antimicrobial classes following the classification system applied by WHO (Fig 2) [8]. Data on cyclic polypeptides, ionophores, nitrofurans, nitroimidazoles and steroid antibacterials are not presented as it would affect business and trade secrets of marketing authorization holders in Germany. Detailed sales volumes of the different antimicrobial classes are provided as supplementary material (S1 File).

Tetracyclines (HIA) and aminopenicillins (CIA) constituted the major part of veterinary antimicrobial sales in Germany. Initial volumes determined for 2011 amounted to 564 tons and 501 tons, respectively. In 2018, both antimicrobial classes still accounted for the highest sales volumes with 178 tons (reduction by 68.38%) and 240 tons (reduction by 52.09%), respectively, but the order had changed in 2014. Further antimicrobial classes initially exceeding 100 tons were sulfonamides and dihydrofolate reductase inhibitors (215 tons), macrolides (173 tons) and polymyxins (127 tons). It was primarily the polymyxins that altered their ranking over time. Initially constituting for the 5^th^ highest sales volumes, polymyxins started to exceed macrolides in 2015 (82 tons vs. 52 tons). In 2017, sales volumes of polymyxins (74 tons) also exceeded those of sulfonamides and dihydrofolate reductase inhibitors (70 tons), accounting for the 3^rd^ highest sales volumes in Germany. Total reductions from 2011 to 2018 amounted to 66.11% for macrolides, 42.21% for polymyxins and 66.78% for sulfonamides and dihydrofolate reductase inhibitors.

It is noted that increases in sales volumes of certain antimicrobial classes were observed from 2015 to 2018. This accounts for aminoglycosides (5 tons or 32.77% increase), amphenicols (1 tonne or 20.10% increase), macrolides (6 tons or 11.84% increase) and narrow spectrum penicillins (4 tons or 16.33% increase). Sales volumes of polymyxins increased by 5 tons or 6.76% from 2016 to 2017. Unlike the recent development of sales volumes of the Highest Priority CIAs macrolides and polymyxins, sales volumes of cephalosporins of the 3^rd^ and 4^th^ generation and of quinolones decreased from 2017 to 2018. This contrasts with the previously observed development of sales volumes of the respective substance classes (Table 4). While sales volumes of cephalosporins of the 4^th^ generation slightly decreased (with the exception of the year 2014), those of cephalosporins of the 3^rd^ generation and quinolones initially increased. Sales volumes of cephalosporins of the 3^rd^ generation peaked in 2012 with an increase of 14.04% referred to the year 2011, those of quinolones in 2014 with an increase of 49.70%. In 2017, sales volumes of cephalosporins of the 3^rd^ generation and of quinolones still exceeded the initial 2011 sales volumes by 13.51% and 20,10%, respectively. From 2017 to 2018, sales volumes of cephalosporins of the 3^rd^ and 4^th^ generation largely decreased by 46.24% and 55.37% respectively. Sales volumes of quinolones were reduced by 722.09% within the same period.

**Table 4:** Sales volumes of Critically Important Antimicrobials of Highest Priority.

	CEP3		CEP4		MCL		PMY		QUI	
year	volume [t]	change [%]	volume [t]	change [%]	volume [t]	change [%]	volume [t]	change [%]	volume [t]	change [%]
2011	2,057		1,427		173,137		127,339		8,247	
2012	2,346	+14,04	1,399	-1,95	144,676	-16,44	123,478	-3,03	10,382	+25,89
2013	2,320	-1,10	1,363	-2,58	126,046	-12,88	124,701	+0,99	12,125	+16,78
2014	2,315	-0,20	1,401	+2,78	108,667	-13,79	106,657	-14,47	12,346	+1,82
2015	2,280	-1,54	1,325	-5,39	52,463	-51,72	81,825	-23,28	10,555	-14,50
2016	2,301	+0,94	1,122	-15,32	54,663	+4,19	68,909	-15,78	9,339	-11,52
2017	2,335	+1,49	1,062	-5,39	54,723	+0,11	73,566	+6,76	9,905	+6,06
2018	1,256	-46,24	0,474	-55,37	58,677	+7,23	73,594	+0,04	7,717	-22,09
**change**	**-0,801**	**-38,96**	**-0,953**	**-66,79**	**-114,460**	**-66,11**	**-53,745**	**-42,21**	**-0,530**	**-6,43**

Sales volumes are expressed in tons of active ingredient [t]. Changes in percent refer to the previous year. CEP3: cephalosporins of the 3^rd^ generation, CEP4: cephalosporins of the 4^th^ generation, MCL: macrolides, PMY: polymyxins, QUI: quinolones

### Sales volumes of active ingredients within the class of tetracyclines

**Fig 3:**
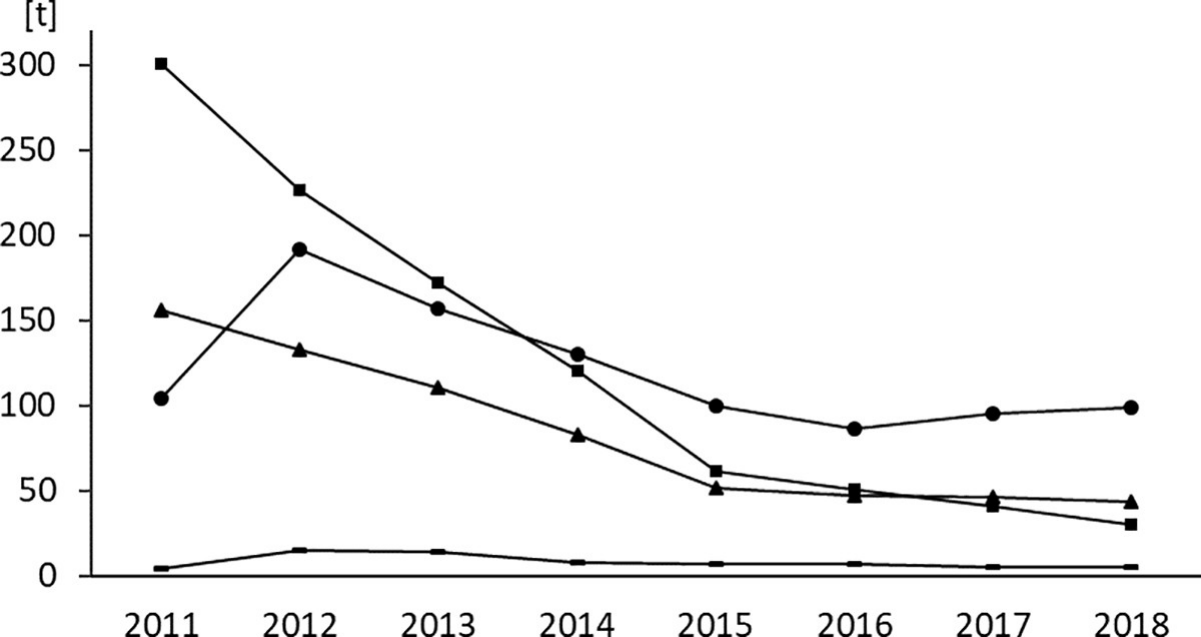
Sales volumes of tetracyclines. Sales volumes are expressed in tons [t] of active ingredient. Squares: tetracycline, triangles: chlortetracycline, circles: doxycycline, dashes: oxytetracycline.

Within the antimicrobial class of tetracyclines, a marked change in sales volumes between the corresponding antimicrobial agents was noted (Fig 3). From 2011 to 2012, total sales volumes of tetracyclines remained nearly unchanged with 564 tons and 566 tons, respectively, while a switch from a predominant sale of tetracycline (300 tons) to a nearly equalized sale of tetracycline and doxycycline (226 tons vs. 192 tons) was determined. From 2014 onwards, doxycycline constituted for the largest share on sales volumes within the class of tetracyclines. In 2018, sales volumes of doxycycline remained only slightly below those of the initial 2011 data (104 tons vs. 99 tons).

## Discussion

A considerable reduction of total sales volumes of antimicrobials for veterinary use was observed by the German surveillance system from 2011 to 2018, amounting to 983 tons (58%). Such large reductions are most likely easier to achieve in countries starting with a comparably high baseline, as it was the case in Germany. Regarding the initial 2011 data on sales of antimicrobial agents for veterinary use in food-producing animals, Germany was accordingly ranked fourth highest behind Cyprus, Italy and Spain by the European Surveillance of Veterinary Antimicrobial Consumption (ESVAC) project with 211.5 mg per population correction unit (PCU). The latest available report refers to the year 2017 and assigned Germany at position twelve with 89 mg/PCU [37]. It should be noted that absolute sales volumes presented in ESVAC reports deviate from our reported national data due to differences in data processing.

Comparisons to the amounts used in human medicine in Germany within the same period are hampered by the fact that to date, nationwide data is only collected for the community sector while comprehensive data on antimicrobial use in hospitals are lacking. From 2011 to 2018, an overall reduction of antimicrobial use in the German community sector by 9.16% was observed, based on the metrics of defined daily doses per 1,000 inhabitants per day [38]. In 2014, total consumption in human medicine was briefly estimated to range between 700 to 800 tons [39]. It is thus assumed that volumes of antimicrobials used in human and veterinary medicine in Germany are currently nearly equal.

In contrast to other European countries like Belgium [40, 41], Denmark [42, 43], France [44], Norway [45], the Netherlands [46–48] and the United Kingdom [49], there has never been a policy setting defined targets for a reduction of sales volumes in veterinary medicine in Germany. Furthermore, no other legally binding measures on veterinary antimicrobial consumption were implemented until 2014. This raises the question why a constant decrease of sales volumes was determined right from the start of the German surveillance. Most likely, this can be attributed to an increasing awareness on the growing burden of antimicrobial resistance of veterinary surgeons, farmers and the society, arising from an augmented focus on the possible contribution of the animal sector. Particularly the occurrence of severe disease courses of livestock-associated (LA)-MRSA infections in humans could have been crucial. They lead to increased media coverage and numerous research publications on a transmission of resistant bacteria and the resulting risk for human health [50–54]. Countries like Denmark and the Netherlands were reported to take early action to control the risk of antimicrobial resistance emerging and spreading from farms, so a spill-over effect to a certain extent is also conceivable. Another reason could be the simple fact that surveillance was conducted. This phenomenon is termed the ‘surveillance effect’. The impact of sales surveillance itself on antimicrobial consumption has never been investigated but the effect was evidenced in other sectors. The participation in surveillance programmes was shown to reduce incidences of nosocomial infections even in the absence of specific prevention actions, just by raising awareness [55–58]. Even though the surveillance of the veterinary sector was not conducted at farm or veterinary practice level in Germany, it is likely to have affected the prescription and use behavior of veterinarians and farmers to a certain extent. The first publication of sales volumes for the year 2011 attracted considerable media attention. A particular focus was drawn to the fact that the initial sales volumes were much higher than previous estimates had suggested, increasing awareness on an indeed high antimicrobial use in animals in Germany.

On the downside, it is likely that a part of the reduction in sales volumes was not attributable to an actual reduction of veterinary antimicrobial use. Factors affecting sales volumes are changes in animal demographics and in prescription patterns [35, 59–63]. Changes in animal demographics are not taken into account by the German system [35]. Reliable data is available on the numbers of food-producing animals in Germany while companion animals are not comprehensively registered. The population of food-producing animals was consistent throughout the investigation period in Germany (PCU of approx. 8,600) [37] and did therefore not affect sales volumes. It can also be ruled out that a part of the reduction was attributable to an increased prescription of medicated premixes which are excluded from the German surveillance system [37]. Sales volumes of medicated premixes in Germany are amongst the lowest of all European countries and consistently accounted for less than 0.5% of total sales volumes throughout the investigation period. The occurrence of increases in sales volumes of certain antimicrobial classes while total sales volumes decrease provides evidence for changes in prescription patterns. This phenomenon was observed for numerous antimicrobial classes from 2011 to 2014, including the highly potent antimicrobial classes of cephalosporins of the 3^rd^ generation and quinolones. Furthermore, a shift within the class of tetracyclines was detected, from tetracycline to the more potent doxycycline. This was substantiated by data retrieved from AMIS indicating the granting of numerous marketing authorizations of products containing doxycycline from 2009 to 2012. However, only the analysis of use data collected at farm or veterinary practice level would allow for a quantification of the impact of changes in prescription patterns on sales or consumption volumes across antimicrobial agents and antimicrobial classes [35, 63–65].

The first legally binding measure for a reduction of antimicrobial consumption in Germany was implemented by the treatment frequencies benchmarking targeting fattening farms for pigs, beef cattle, broiler chickens and turkeys [33]. Farms whose individual treatment frequency exceeds the median of the treatment frequencies of all farms of the same production type, have to evaluate the causes for their frequent antimicrobial use under consultation of a veterinarian and take appropriate measures for future reduction. If the 3^rd^ quartile is also exceeded, a written action plan has to be provided for assessment by the competent authority. The occurrence of the highest annual reduction in German sales volumes by 35% that was observed from 2014 to 2015, was most likely attributable to the introduction of the benchmarking in 2014. Antimicrobial classes that contributed most to this decrease were those widely used in food-producing animals—aminoglycosides, macrolides, amino- and narrow spectrum penicillins, sulfonamides and dihydrofolate reductase inhibitors as well as tetracyclines. Similarities to the development of sales or consumption volumes of other countries after implementation of a benchmarking system were apparent. The first year after establishment of the Yellow Card Initiative for pig farms in Denmark in 2010, a pronounced decrease in consumption of antimicrobial agents for veterinary use was observed, followed by a slight increase until 2013 [42, 43]. In the Netherlands, a benchmarking system targeting livestock farms was implemented in 2011, leading to a pronounced decrease in sales volumes from 2010 to 2012 that lessened thereafter [66]. Regarding Germany, the reduction of sales volumes also decelerated as of the second year after introduction of the benchmarking, amounting to 10% in total from 2015 to 2018. In 2019, the impact of the treatment frequencies benchmarking was evaluated by the Federal Ministry of Food and Agriculture [67]. While the analysis of sales volumes indicated a strong impact, the investigation of use data collected at farm level as part of the evaluation revealed substantial differences between the targeted production types. From 2014 to 2015, a strong reduction of antimicrobial consumption in pig production was determined while that of the other sectors decreased only slightly (cattle) or remained nearly unchanged (poultry). Since most veterinary antimicrobial products are authorized for use in various animal species [35, 61, 63, 65], such differences occurring at species or production type level were not detectable by an assessment of sales volumes.

WHO developed the CIA list upon recommendation of the Tripartite (WHO/Food and Agriculture Organization of the United Nations (FAO)/World Organisation for Animal Health (OIE)), intended to aid in risk management of antimicrobial resistance due to the non-human use of antimicrobials [68]. Advice provided by WHO and the Tripartite on the veterinary use of antimicrobials in accordance with the prioritization scheme mainly concerned the sector of animal production [23, 69, 70], but should also apply to companion animals wherever possible, due to the risk of a transmission of resistance via direct contact to humans [24, 71, 72]. WHO recommended in its ‘Guidelines on use of medically important antimicrobials in food-producing animals’ the preferred use of antimicrobials considered of least importance for human health while any use of CIAs of Highest Priority should be justified by susceptibility testing, indicating the respective antimicrobial as the only treatment option [73]. Following the precautionary principle, it was conditionally recommended to not use CIAs for metaphylactic treatments and to cease the use of CIAs of Highest Priority in food animals. Regarding Germany, compliance with the prioritization of antimicrobials and the resulting recommendations has to be considered low. In Denmark, Finland, the Netherlands and the UK, different animal or food production sectors voluntarily decided upon a restriction or cessation of certain compounds belonging to the category of CIAs of Highest Priority [74]. However, such initiatives are absent in Germany. Although absolute sales volumes of CIAs were largely reduced, they still accounted for the highest proportion in Germany in 2018. However, differences in compliance between the various veterinary sectors with respect to species and, concerning the food animal sector, also production and farm type may be present that cannot be detected by a surveillance system based on sales volumes. Besides, compliance with the WHO prioritization might not always be feasible. OIE was requested by the Tripartite to develop a list similar to that of WHO, prioritizing antimicrobials according to their relevance for veterinary medicine. Antimicrobial classes categorized as Veterinary Critically Important Antimicrobials can thus be the only available treatment option for specific severe infectious diseases in certain animal species [6]. Common indications for the use of CIAs of Highest Priority, for which sufficient treatment alternatives can be lacking in some instances, are enteric and respiratory infections in pigs, cattle and poultry, urogenital infections in cats and dogs and respiratory infections in horses [75]. However, even in such cases a potential for a reduction of antimicrobial use is seen by taking preventive measures, for instance vaccination and improvement of housing conditions.

Similar to the situation regarding total sales volumes, Germany has never defined specific targets for the reduction of sales volumes of antimicrobial classes belonging to the subcategory of CIAs of Highest Priority either, in contrast to other European countries like Belgium [40, 41], France [44] and the Netherlands [46–48]. Until 2018, no legally binding measures targeting CIAs of Highest Priority were implemented, whereas susceptibility testing had been made obligatory in Denmark, France, the Netherlands and Czech Republic, factors for differentiated taxes had been established in Belgium and the use of the cascade (a stepwise approach allowing for an off-label use of veterinary and human medicinal products under defined circumstances) had been restricted in Finland [74]. Overall, contradictory developments were observed regarding sales volumes of CIAs of Highest Priority in Germany. While sales volumes of macrolides and polymyxins largely reduced at first but increased again the recent years, sales volumes of cephalosporins of the 3^rd^ generation and of quinolones initially increased and still amounted to higher volumes in 2017 compared to the 2011 data. On March 1^st^, 2018 the ‘2^nd^ Amendment of the Veterinary Pharmacies Prescription Regulation (TÄHAV)’ came into force, addressing the issue of antimicrobial resistance by an optimization of therapy [76]. In this context, susceptibility testing was made obligatory for cephalosporins of the 3^rd^ and 4^th^ generation and for quinolones. The analysis of sales volumes of the respective antimicrobial classes for 2018 showed a marked impact of the measure on cephalosporins of the 3^rd^ and 4^th^ generation that was much less pronounced on quinolones. Increased marketing efforts were undertaken by some marketing authorization holders for veterinary medicinal products containing fluoroquinolones shortly after the amendment of the regulation came into force, but might have been conducted for products containing cephalosporins of the 3^rd^ or 4^th^ generation as well. Without the provision of use data, the situation cannot be profoundly evaluated [63, 65, 77].

Judging from the strong underestimation of sales volumes of antimicrobials for veterinary use in Germany until 2011, the implementation of a comprehensive surveillance system constituted a vital step to gain reliable knowledge on antimicrobial volumes in veterinary medicine. Moreover, the conduction of sales surveillance has to generally be considered essential for data retrieval on a global scale and for inter-country comparison [78]. For this purpose, standardized procedures are required that can also be adopted by low- and middle-income countries [63, 79]. Sales surveillance has to be regarded the simplest way to gather data and is thus the easiest to implement [78–80]. A drawback of the German sales surveillance system is the exclusion of off-label use of human antimicrobial products. This is of special concern regarding antimicrobial classes like carbapenems that are currently not authorized as veterinary medicinal products but listed as CIAs by WHO. Carbapenem-resistant strains of Enterobacteriaceae isolated from animals were reported in Germany, mainly originating from dogs at a university veterinary clinic [81]. The use of such antimicrobial classes is hampered in food-producing animals by the requirement of establishment of maximum residue limits [82], but off-label use is possible in companion animals in accordance with the German cascade under defined circumstances [33]. To which extent and in which context they are actually administered is widely unknown but requires investigation [83, 84]. The extent could be determined via collection of sales data by the current German surveillance system if it would be accordingly optimized. However, questions on the context of use with respect to animal species and indications would remain unanswered as they do for all antimicrobial classes, independent of existing authorizations for veterinary medicine.

The conduction of sales surveillance can only be considered an initial step for the surveillance of antimicrobials in veterinary medicine. If a country proceeds to implement risk management measures, areas at highest risk need to be identified for a targeted deduction [79, 80, 85, 86]. Once measures are established, they require evaluation to ensure appropriateness and exertion of the desired impact [79, 85]. The capability of the German surveillance system based on sales volumes was shown to be limited in both regards since the collected data cannot be disaggregated according to animal species, production types and indications for treatment. Stratified data would as well be required for the establishment of an evaluation system based on defined daily doses (DDD) or used daily doses (UDD), which is thus not feasible by the German sales surveillance system [87, 88].

It has to be borne in mind that all measures intended to promote a prudent use or reduce antimicrobial consumption in animals pursue the goal to control the risk of antimicrobial resistance emerging from the veterinary use of antimicrobials. As part of the evaluation of the German treatment frequencies benchmarking, potential impacts on antimicrobial resistance were investigated, using, inter alia, clinical *E*. *coli* isolates as indicator [67]. While prevalence of resistance in isolates originating from broiler chickens, piglets and young cattle mainly remained unchanged or slightly decreased from 2009 to 2017, prevalence of Cefotaxim resistance of isolates from calves and Enrofloxacin resistance of isolates from turkeys increased. Reliable use quantities of the respective antimicrobial classes according to animal species and indications would be required to directly relate antimicrobial consumption to this development, taking the known differences in resistance prevalence of animal pathogenic bacteria in between species and indications into account [39, 89]. The evaluation of impacts of antimicrobial consumption on antimicrobial resistance is thus not feasible by a surveillance system collecting sales data at the level of pharmaceutical companies and wholesalers.

Based upon the numerous drawbacks of the German surveillance system that were outlined above, the required progress in surveillance can only be achieved by establishment of a surveillance system collecting use data at the level of farms or veterinary practices. The EU is currently experiencing a major change in legislation in this regard. The Regulation (EU) 2019/6 on veterinary medicinal products [90] that will be applicable in all Member States as of January 28^th^, 2022 stipulates the stepwise implementation of use data collection of antimicrobial agents in addition to the collection of sales data (Article 57). Types of comprised antimicrobial products as well as rules on methods for data gathering and quality assurance are currently established by means of a delegated act. The data gaps of the German surveillance system will thus be filled in the future but not until interim periods have passed. The reporting of use data of major species of food-producing animals will be due latest in 2024 and that of companion animals latest in 2030. It is advised to start collecting data for the concerned species at least one year beforehand [91]. The regulation also provides the legal base for an exclusive reservation of antimicrobial agents for human medicine (Article 37) which would render all veterinary authorizations of the respective antimicrobial agents or classes in all European Member States invalid and prohibit a future veterinary authorization [90]. Furthermore, the Commission may establish lists of antimicrobial agents that are subject to restrictions for off-label use under the cascade (Article 107) [90].

## Conclusion

The evaluation of data collected by the German sales surveillance system for eight consecutive years determined a considerable reduction of sales volumes of antimicrobial agents for veterinary use by 58%. The reduction was achieved without a setting of legal targets but via surveillance and legal implementation of a benchmarking system for fattening farms. In contrast to the general demand for reduction of antimicrobial use in animals, compliance with the particular demand for a reduction of CIAs of Highest Priority was shown to be low in Germany without enforcement by specific regulatory interventions. Only the stipulation of susceptibility testing for cephalosporins of the 3^rd^ and 4^th^ generation and fluoroquinolones lead to a considerable reduction of sales volumes of the respective antimicrobial classes.

A surveillance system collecting sales data at the level of pharmaceutical companies and wholesalers has to be considered an initial step to gain knowledge on antimicrobial volumes in veterinary medicine, especially regarding the retrieval of data on a global scale. However, its usability for risk management of antimicrobial resistance is limited. To progress by identifying areas at highest risk for a targeted deduction of measures on antimicrobial use in animals and to subsequently evaluate their impact and appropriateness, a surveillance system based on use data is required. It should be combined or even harmonized with effective monitoring programs investigating the development of antimicrobial resistance.

## Supporting information

S1 File (DOCX)
