# Supplementary material for: Eight years of sales surveillance of antimicrobials for veterinary use in Germany—What are the perceptions?
Source: PLoS One. 2020 Aug 10;15(8):e0237459. doi: 10.1371/journal.pone.0237459 (PMC7416935; doi:10.1371/journal.pone.0237459)
Supplement: S1 File — (DOCX) [file pone.0237459.s001.docx]

| **Sales volumes of antimicrobial classes in Germany expressed in tonnes according to the categorization of substance classes of 6^th^ revision of the WHO CIA list** | | | | | | | | |  |  |  |
| --- | --- | --- | --- | --- | --- | --- | --- | --- | --- | --- | --- |
| total changes refers to the developments from 2011 to 2018 | |  |  |  |  |  |  |  |  |  |  |
| * detailed data are not presented as it would affect business and trade secrets of marketing authorization holders in Germany | | | | | | |  |  |  |  |  |
| ^#^ not authorized for use in human medicine |  |  |  |  |  |  |  |  |  |  |  |
|  |  |  |  |  |  |  |  |  |  |  |  |
| **year** | **2011** | **2012** | **2013** | **2014** | **2015** | **2016** | **2017** | **2018** |  | **total change [t]** | **total change [%]** |
|  |  |  |  |  |  |  |  |  |  |  |  |
| ***Important Antimcirobials (IA)*** |  |  |  |  |  |  |  |  |  |  |  |
| aminocyclitols | 9.262 | 9.375 | 11.315 | 11.416 | 8.590 | 8.085 | 11.048 | 8.131 |  | -1.131 | -12.21 |
| cyclic polypeptides* |  |  |  |  |  |  |  |  |  |  |  |
| ionophores*^#^ |  |  |  |  |  |  |  |  |  |  |  |
| nitrofurans* |  |  |  |  |  |  |  |  |  |  |  |
| nitroimidazoles* |  |  |  |  |  |  |  |  |  |  |  |
| pleuromutilins | 14.101 | 18.362 | 15.456 | 12.978 | 11.218 | 9.944 | 13.374 | 8.233 |  | -5.868 | -41.61 |
| **total sales volumes of IA** | **23.500** | **27.861** | **28.668** | **26.875** | **20.130** | **20.826** | **27.831** | **20.277** |  | **-3.223** | **-13.71** |
|  |  |  |  |  |  |  |  |  |  |  |  |
| ***Highly Important Antimicrobials (HIA)*** |  |  |  |  |  |  |  |  |  |  |  |
| amphenicols | 6.119 | 5.704 | 5.230 | 5.273 | 5.026 | 5.121 | 5.577 | 6.036 |  | -0.083 | -1.36 |
| cephalosporins of the 1^st^ generation* |  |  |  |  |  |  |  |  |  |  |  |
| lincosamides | 16.809 | 15.389 | 16.896 | 14.614 | 10.769 | 9.877 | 10.857 | 9.910 |  | -6.899 | -41.04 |
| anti-staphylococcal penicillins | 4.083 | 4.465 | 4.030 | 4.555 | 3.963 | 3.480 | 3.072 | 2.806 |  | -1.277 | -31.28 |
| narrow spectrum penicillins | 22.460 | 42.087 | 44.627 | 36.659 | 24.206 | 24.477 | 26.159 | 28.160 |  | 5.700 | 25.38 |
| steroid antibiotics* |  |  |  |  |  |  |  |  |  |  |  |
| sulfonamides and dihydrofolate reductase inhibitors | 214.739 | 188.011 | 176.513 | 140.113 | 82.881 | 78.555 | 70.207 | 71.338 |  | -143.401 | -66.78 |
| tetracyclines | 564.429 | 566.162 | 454.435 | 341.849 | 220.530 | 192.550 | 187.753 | 178.498 |  | -385.931 | -68.38 |
| **total sales volumes of HIA** | **830.674** | **823.886** | **703.794** | **545.143** | **349.329** | **316.029** | **305.607** | **298.860** |  | **-531.814** | **-64.02** |
|  |  |  |  |  |  |  |  |  |  |  |  |

| ***Critically Important Antimicrobials (CIA)*** |  |  |  |  |  |  |  |  |  |  |  |
| --- | --- | --- | --- | --- | --- | --- | --- | --- | --- | --- | --- |
|  |  |  |  |  |  |  |  |  |  |  |  |
| *CIA of High Priority* |  |  |  |  |  |  |  |  |  |  |  |
| aminoglycosides | 37.883 | 30.870 | 28.056 | 26.360 | 16.097 | 18.055 | 18.255 | 21.373 |  | -16.510 | -43.58 |
| aminopenicllins | 501.395 | 454.141 | 424.546 | 408.576 | 271.277 | 251.013 | 239.824 | 240.202 |  | -261.193 | -52.09 |
| total sales volumes of CIA of High Priority | 539.278 | 485.011 | 452.602 | 434.937 | 287.374 | 269.068 | 258.079 | 261.575 |  | -277.703 | -51.50 |
|  |  |  |  |  |  |  |  |  |  |  |  |
| *CIA of Highest Priority* |  |  |  |  |  |  |  |  |  |  |  |
| cephalosporins of the 3^rd^ generation | 2.057 | 2.346 | 2.320 | 2.315 | 2.280 | 2.301 | 2.335 | 1.256 |  | -0.801 | -38.94 |
| cephalosporins of the 4^th^ generation | 1.427 | 1.399 | 1.363 | 1.401 | 1.325 | 1.122 | 1.062 | 0.474 |  | -0.953 | -66.78 |
| macrolides | 173.137 | 144.676 | 126.046 | 108.667 | 52.463 | 54.663 | 54.723 | 58.677 |  | -114.460 | -66.11 |
| polymyxins | 127.339 | 123.478 | 124.701 | 106.657 | 81.825 | 68.909 | 73.566 | 73.594 |  | -53.745 | -42.21 |
| quinolones | 8.247 | 10.382 | 12.125 | 12.346 | 10.555 | 9.339 | 9.905 | 7.717 |  | -0.530 | -6.43 |
| total sales volumes of CIA of Highest Priority | 312.207 | 282.281 | 266.555 | 231.385 | 148.448 | 136.335 | 141.591 | 141.718 |  | -170.489 | -54.61 |
|  |  |  |  |  |  |  |  |  |  |  |  |
| **total sales volumes of CIA** | **851.485** | **767.292** | **719.157** | **666.322** | **435.822** | **405.403** | **399.670** | **403.293** |  | **-448.192** | **-52.64** |
|  |  |  |  |  |  |  |  |  |  |  |  |
| **total sales volumes of all antimicrobial classes** | **1705.659** | **1619.039** | **1451.619** | **1238.340** | **805.281** | **742.258** | **733.108** | **722.430** |  | **-983.229** | **-57.65** |
